# Optimal Length of Heart Rate Variability Data and Forecasting Time for Ventricular Fibrillation Prediction Using Machine Learning

**DOI:** 10.1155/2021/6663996

**Published:** 2021-03-16

**Authors:** Da Un Jeong, Getu Tadele Taye, Han-Jeong Hwang, Ki Moo Lim

**Affiliations:** ^1^Department of IT Convergence Engineering, Kumoh National Institute of Technology, Gumi 39177, Republic of Korea; ^2^Health Informatics Units, School of Public Health, Mekelle University, Mekelle, Ethiopia; ^3^Department of Electronics and Information Engineering, Korea University, Sejong, Republic of Korea; ^4^Department of Medical IT Convergence Engineering, Kumoh National Institute of Technology, Gumi 39177, Republic of Korea

## Abstract

Ventricular fibrillation (VF) is a cardiovascular disease that is one of the major causes of mortality worldwide, according to the World Health Organization. Heart rate variability (HRV) is a biomarker that is used for detecting and predicting life-threatening arrhythmias. Predicting the occurrence of VF in advance is important for saving patients from sudden death. We extracted features from seven HRV data lengths to predict the onset of VF before nine different forecast times and observed the prediction accuracies. By using only five features, an artificial neural network classifier was trained and validated based on 10-fold cross-validation. Maximum prediction accuracies of 88.18% and 88.64% were observed at HRV data lengths of 10 and 20 s, respectively, at a forecast time of 0 s. The worst prediction accuracy was recorded at an HRV data length of 70 s and a forecast time of 80 s. Our results showed that features extracted from HRV signals near the VF onset could yield relatively high VF prediction accuracies.

## 1. Introduction

Cardiovascular disease (CVD) is the leading cause of death in many developed countries [1] and accounts for an estimated 31% of all global deaths according to the World Health Organization, making it the primary cause of mortality worldwide. CVD includes abnormal heart rhythms, called ventricular tachyarrhythmias (VTAs), which comprise ventricular fibrillation (VF) and ventricular tachycardia (VT). VTA is one of the factors causing a fast heart rate and mortality in the absence of immediate medical treatment [2]. Therefore, early prediction of VTA is important to save patients from sudden death. The development of early preventive interventions could reduce the risk of an imminent VTA event.

Heart rate variability (HRV) indices have been used as noninvasive biomarkers to predict life-threatening VTAs such as VF [3]. These indices provide features useful for distinguishing arrhythmia from normal HRV [4]. HRV signifies the time variation of the R-peaks between two successive QRS complexes. The maximum upward deflection of a normal QRS complex is the R-peak, and the duration between two consecutive R-peaks is called the RR interval [5]. Previous studies have analyzed HRV by quantifying its features using three analysis methods: time-domain, frequency-domain, and Poincare nonlinear analyses [2, 6, 7].

Several studies have used the above three HRV analysis methods to analyze short-term (~5 min) and ultra-short-term (<5 min) HRV data. Short-term analysis has been widely considered to be methodologically reliable for analyzing HRV data [8] and for extracting features to investigate the occurrence of VF. Joo et al. applied an artificial neural network (ANN) to predict VTAs (both VT and VF) 10 s before their occurrence, using short-term HRV data [6]. Recently, Lee et al. attempted to predict the occurrence of VT using HRV features extracted from short-term HRV data [2]. Although short-term HRV data showed promising performance in predicting VTAs, ultra-short-term HRV data were found to be adequate for personal health devices with limited performance and memory capacity [9]. Therefore, recent studies have focused on ultra-short-term HRV analysis for applications in ambulatory heart rate devices [9]. Ebrahimzadeh et al. used the time-frequency and Poincare nonlinear analyses to extract HRV features from ultra-short-term HRV data. They extracted features from different segments of the HRV signals at successive 1 min ultra-short-term HRV intervals (i.e., at the first, second, third, and fourth minute before the event). They used the multilayer perceptron and *K*-nearest neighbor algorithms to classify healthy subjects and patients with VTA [10]. Finally, they compared the accuracies for predicting the occurrence of VTA by employing various time-series forecasting models with a fixed data length (1 min).

Although several studies have attempted to use ultra-short-term HRV data, the optimal data length and forecast time period have yet to be determined. Therefore, in this study, multiple combinations of different HRV data lengths and forecast time periods were investigated to obtain the optimal HRV data and forecast time interval for our data set. The objective of this study was to investigate the optimal HRV data lengths and forecast time periods for VF prediction, by comparing the performance in VF prediction of various HRV data lengths and forecast time periods. We evaluated nine different forecast times (from 0 to 80 s at 10 s intervals) and seven different HRV data lengths (from 10 to 70 s at 10 s intervals). All combinations of different HRV data lengths and forecast time periods were assessed based on the prediction accuracies obtained using the ANN algorithm.

## 2. Materials and Methods

### 2.1. Database

We used the following databases from PhysioNet [11]: Creighton University VTA database (CUDB) [12], spontaneous VTA database (MVTDB) [13], normal data sets from the paroxysmal atrial fibrillation prediction challenge database (PAFDB) [14], and the Massachusetts Institute of Technology-Beth Israel Hospital normal sinus rhythm database (NSRDB) [11]. We selected 29 VF subjects from CUDB, 29 VF and 30 control subjects from MVTDB, and 30 control subjects from PAFDB and NSRDB (12 and 18 subjects, respectively), resulting in a total of 58 VF subjects and 60 control subjects. The sampling rates were 250 Hz for CUDB, 1000 Hz for MVTDB, and 128 Hz for the other two data sets.

### 2.2. Preprocessing

**Figure 1:**
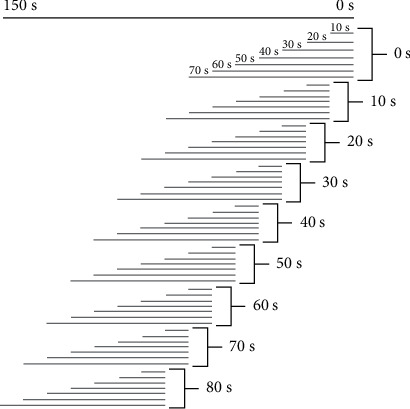
Representation of the seven data lengths from 10 s to 70 s in an interval of 10 s and nine forecast time periods from 0 s to 80 s in an interval of 10 s selected from the total period 150 s.

RR intervals were collected from the PhysioBank Automated Teller Machine, a web service that contains waveforms annotated by cardiologists, for the data used in this study.  Figure 1 shows the procedure used to organize the data sets using different data lengths and forecast time periods. Based on the 150 s HRV signal before the occurrence of VF, we considered nine different forecast time periods ranging from 0 to 80 s at intervals of 10 s. Each forecast time contains seven different HRV data lengths from 10 to 70 s at intervals of 10 s, resulting in 63 combinations. The HRV data length represents the time period used for feature extraction, shown as a gray line in Figure 1. The forecast time is the time period before the VF onset (Figure 1).

### 2.3. Feature Extraction

The features listed in Table 1 were calculated for each HRV data length denoted by the gray segments in Figure 1. These features consist of five HRV features (two features in the time-domain analysis and three features obtained using Poincare nonlinear analysis), which were extracted from each of the 58 VF and 60 control data sets. All HRV features were computed from successive RR intervals.

#### 2.3.1. Time-Domain Features

The time-domain features can be characterized by (1) the mean RR intervals (mean NN [RR]) and (2) the square root of the mean squared difference of the successive NN (RR) interval (RMSSD), which can be defined as follows:

(1) $$\begin{array}{c} \text{Mean NN}=\frac{1}{N}\sum \text{R}\text{R}\left(i\right), \end{array}$$

(2) $$\begin{array}{c} \text{RMSSD}=\sqrt{\frac{1}{N}\sum {\left(\text{RR}\left(i+1\right)-\text{RR}\left(i\right)\right)}^{2}}. \end{array}$$

#### 2.3.2. Poincare Nonlinear Features

The features were a dispersion of the points perpendicular to and along the axis of the identity. Here, SD1, which represents the standard deviation of the points perpendicular to the axis of the line of identity, and SD2, which represents the standard deviation of the points along the axis of identity, were both calculated using Equations (3) and (4). The ratio of SD1 to SD2 was also calculated.

(3) $$\begin{array}{c} \text{SD}1=\sqrt{\frac{1}{2}\text{Var}\left(\text{RR}\left(i\right)-\text{RR}\left(i+1\right)\right)}, \end{array}$$

(4) $$\begin{array}{c} \text{SD}2=\sqrt{2\text{SDN}{\text{N}}^{2}-\frac{1}{2}\text{ SD}1^{2}}. \end{array}$$

### 2.4. Proposed Methods

**Figure 2:**
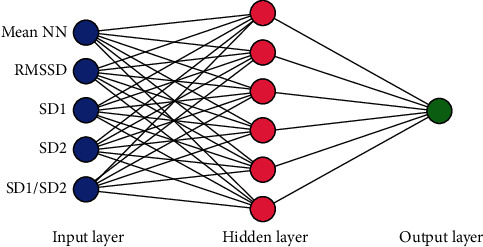
The architecture of our artificial neural network (ANN). The input features to the ANN are as follows: mean NN: mean normal R-peak to normal R-peak interval; SDNN: standard deviation of NN; RMSSD: square root of the mean squared difference of successive NN; pNN50: proportion of the interval differences of successive NN intervals greater than 50 ms; VLF: very low frequency; LF: low frequency; HF: high frequency; SD1: standard deviation of the points perpendicular to the axis of the line of identity; SD2: standard deviation of the points along the axis of identity.

We implemented a fully connected ANN with three layers: an input layer containing input features to the network, a hidden layer capturing the nonlinearity of the data, and an output layer representing the dependent variable (Figure 2) [15, 16]. Rectified linear unit [17] activation functions were used in the input and hidden layers, and a sigmoid activation function [18] was used in the output layer. Through trial and error, six neurons were selected for the hidden layer. All features in the frequency-domain analysis and a few features in the time-domain analysis require a longer recording period to be considered reliable features [8]. Thus, we considered only five such features in this study. The input features were standardized and shuffled before they were used in the ANN. We used a 10-fold cross-validation to avoid overfitting of the classification. The data set was randomly divided into approximately 10 groups: 1 group was used as the testing data set and the remaining 9 groups were used as the training data set. The cross-validation was repeated 10 times to obtain unbiased prediction accuracy. Thus, the final prediction accuracies were determined by estimating the mean and standard deviation of the 10 × 10-fold cross-validation results.

## 3. Results

**Figure 3:**
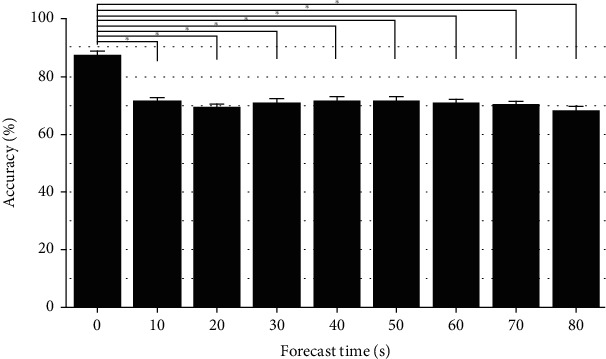
Average accuracies obtained when using the same forecast times over different data lengths. The asterisk (∗) indicates a statistically significant difference between the corresponding groups.

Figure 3 presents the prediction accuracies of all combinations of data length and forecast time. All data lengths with a 0 s forecast time have relatively higher prediction accuracies, indicating that the features extracted from the vicinity of VF onset could well distinguish VF from the control. Furthermore, maximum prediction accuracies of 88.18% and 88.64% were obtained with data lengths of 10 and 20 s, respectively, at a forecast time of 0 s, whereas a minimum prediction accuracy of 64.36% was obtained with a data length of 70 s at a forecast time of 80 s.

**Figure 4:**
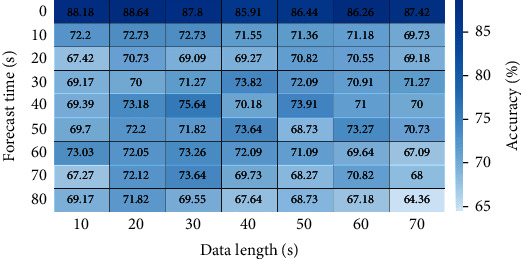
Prediction accuracies of all combinations of the data length and forecast time (63 cases).

Figure 4 shows the mean prediction accuracies with their standard deviations in terms of the forecast time. The highest mean accuracy of 88.75% was obtained when using a 0 s forecast time, which was statistically higher than the accuracies of the other forecast times (analysis of variance [ANOVA] Tukey statistical test, *F*[8, 54] = 68.61, *p* < 0.001), whereas no significant difference was found between the mean prediction accuracies of the other forecast times (*p* > 0.05).

**Figure 5:**
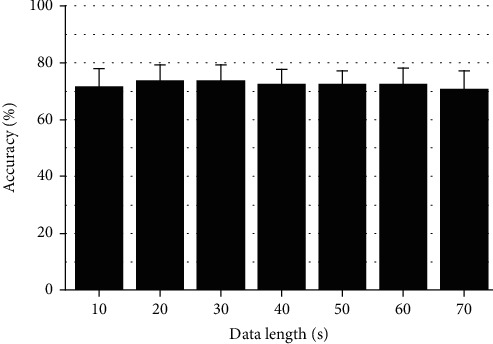
Average accuracies obtained when using the same data lengths over different forecast times.

Figure 5 presents the mean prediction accuracies with their standard deviations in terms of the data length. The ANOVA Tukey statistical test yielded no statistical differences among the mean accuracies for different data lengths.

## 4. Discussion

Advanced technologies have enabled the real-time monitoring of various health conditions by exchanging information between patients and common practitioners. These technologies have inherently low memory and capacity. Therefore, the application of ultra-short-term HRV is inevitably important to analyze HRV in appliances and devices with low memory and capacity [8]. Several studies have investigated ultra-short-term HRV recordings ranging from 10 s to 2 min to determine the reliability of the computed HRV parameters [8, 19]. Although ultra-short-term HRV data are not always reliable for analysis, previous studies have strongly suggested that researchers should consider statistical methods to compensate for the considerable measurement errors caused by the very short HRV segments [19]. Therefore, in this study, we investigated several combinations of data lengths and forecast time periods. Features were extracted from seven different HRV data lengths, each with nine different forecast times, to predict the occurrence of VF in 63 cases. We trained an ANN using these features, and high prediction accuracies were obtained using a forecast time of 0 s. However, the forecast time period of 80 s yielded low prediction accuracies, as shown in Figure 4. The overall results highlighted that features extracted from HRV signals near the VF onset have a higher probability of predicting the occurrence of VF.

We extracted five features using two analysis methods, namely, the time-domain and Poincare nonlinear analysis techniques. However, the frequency-domain features were not considered in this study, as their reliability depends on the length of the HRV signals they are extracted from. All features in the frequency-domain analysis and two features (SDNN and pNN50) in the time-domain analysis require a longer recording period to be considered reliable [8]. Thus, we considered only five features in this study, namely, mean NN, RMSSD, SD1, SD2, and SD1/SD2.

A limitation of this study was that only a few data sets (119 recordings in each case) were used to train our ANN. The ANN model must be trained using more data sets to achieve clinical validation. Further studies involving larger data sets should be conducted to investigate different clinical applications. Finally, the results of this study could be used with a patient's implantable cardiac defibrillator for real-time VF predictions, thus providing additional functionality for VF detection. Predicting the occurrence of VF hours in advance would be particularly useful; however, the data sets used in this study were limited to the 120 s data window and predicted VF 30 s before its occurrence.

## 5. Conclusions

In this study, we trained an ANN to predict VF using features extracted from 63 HRV segments with variable combinations of forecast time periods and data lengths. Subsequently, we determined the optimal data lengths to be 10 and 20 s with a forecast time period of 0 s, which were used to predict the occurrence of VF with relatively high prediction accuracies of 88.18% and 88.64%, respectively. This study could improve the prediction of imminent VF using very short HRV signals.

## Figures and Tables

**Table 1:** Features extracted from the HRV, the QRS complex singed area, and the R-peak amplitude.

Component	Analysis	Feature	Unit	Description
HRV	Time-domain analysis	Mean NN	ms	Mean of normal R-peak to normal R-peak (NN) interval
		RMSSD	ms	Square root of the mean squared differences of successive NN intervals
	Poincare nonlinear analysis	SD1	ms	Standard deviation of points perpendicular to the axis of the line of identity
		SD2	ms	Standard deviation of points along the axis of the line of identity,
		SD1/SD2		Ratio of SD1 over SD2

## Data Availability

The method and result data used to support the findings of this study are included in the article.

## Supplementary Materials

Supplementary Materials The supplementary materials for this article can be found here: Supplementary Table S1 and Table S2 show the summary of the ANN model according to the data length with 0 s of forecast time. Supplementary figures show the features that were used in this study, as the form of heat map graph.
