# Supplementary material for: Optimal Length of Heart Rate Variability Data and Forecasting Time for Ventricular Fibrillation Prediction Using Machine Learning
Source: Comput Math Methods Med. 2021 Mar 16;2021:6663996. doi: 10.1155/2021/6663996 (PMC10435312; doi:10.1155/2021/6663996)
Supplement: Supplementary Materials — The supplementary materials for this article can be found here: Supplementary Table S1 and Table S2 show the summary of the ANN model according to the data length with 0 s of forecast time. Supplementary figures show the features that were used in this study, as the form of heat map graph. [file 6663996.f1.pdf]

Computational and Mathematical Methods in Medicine

Optimal Length of Heart Rate Variability Data and Forecasting Time for Ventricular Fibrillation Prediction Using Machine Learning

Da Un Jeong[1], Getu Tadele Taye[3], Han-Jeong Hwang[4], and Ki Moo Lim[1,2*]

[1] Department of IT Convergence Engineering, Kumoh National Institute of Technology, Gumi 39177, Republic of Korea.
[2] Department of Medical IT Convergence Engineering, Kumoh National Institute of Technology, Gumi 39177, Republic of Korea.
[3]Health Informatics Units, School of Public Health, Mekelle University, Mekelle Ethiopia
[4]Department of Electronics and Information Engineering, Korea University, Sejong, Republic of Korea

*Correspondence should be addressed to Ki Moo Lim; kmlim@kumoh.ac.kr

Supplementary Tables

Supplementary Table S 1. Summary of ANN model validation using MVTDB dataset of 10s data length with 0s of forecast time.

	Precision	Recall	F1-score	Accuracy
VF	0.74	1.00	0.85	0.83
Normal	1.00	0.67	0.80	
Average	0.87	0.83	0.82	

Supplementary Table S 2. Summary of ANN model validation using MVTDB dataset of 20s data length with 0s of forecast time.

	Precision	Recall	F1-score	Accuracy
VF	0.78	1.00	0.88	0.86
Normal	1.00	0.71	0.83	
Average	0.89	0.86	0.85	

Supplementary Figures

The features that were used in this study are provided in the form of heatmap graph in this section. Datasets for the VF and Control datasets are indicated by red and blue line, respectively.

Time domain features for HRV

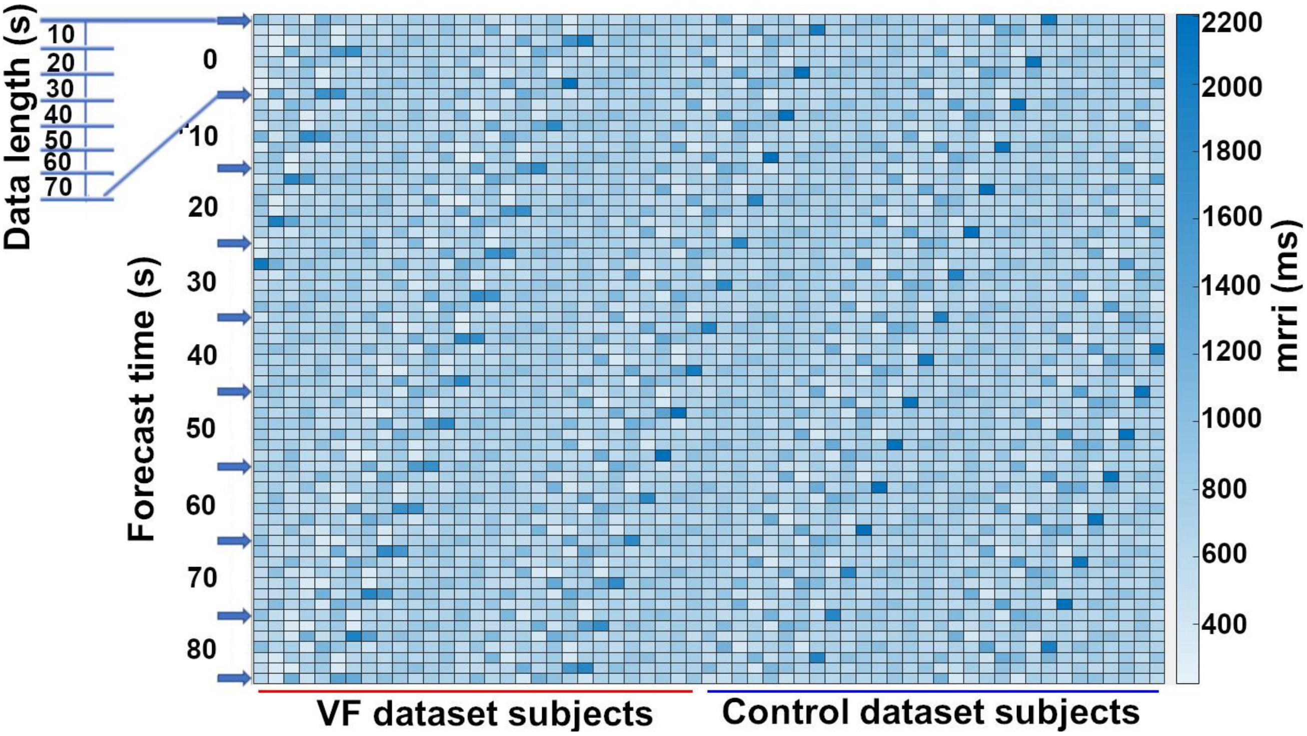

Figure 1. Mean of normal R-peak to normal R-peak (RR) interval (mrri).

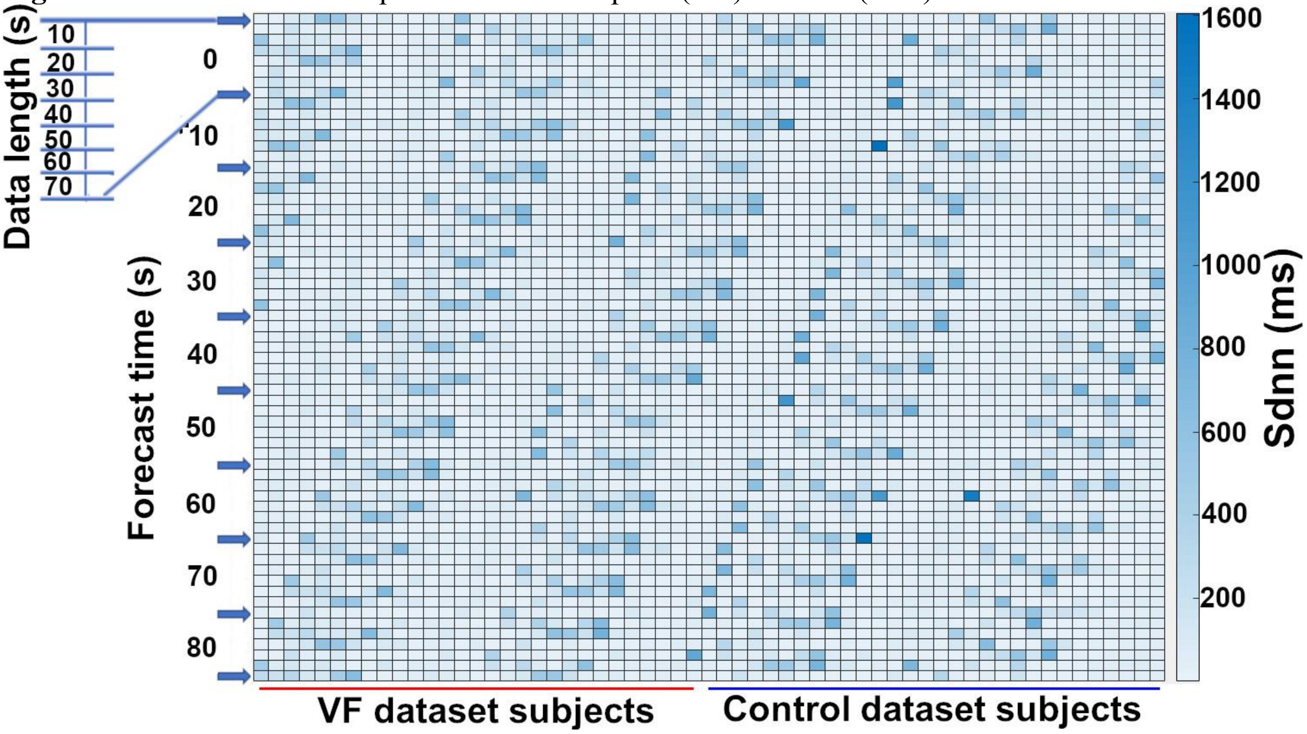

Figure 2. Standard deviation of RR intervals (sdnn).

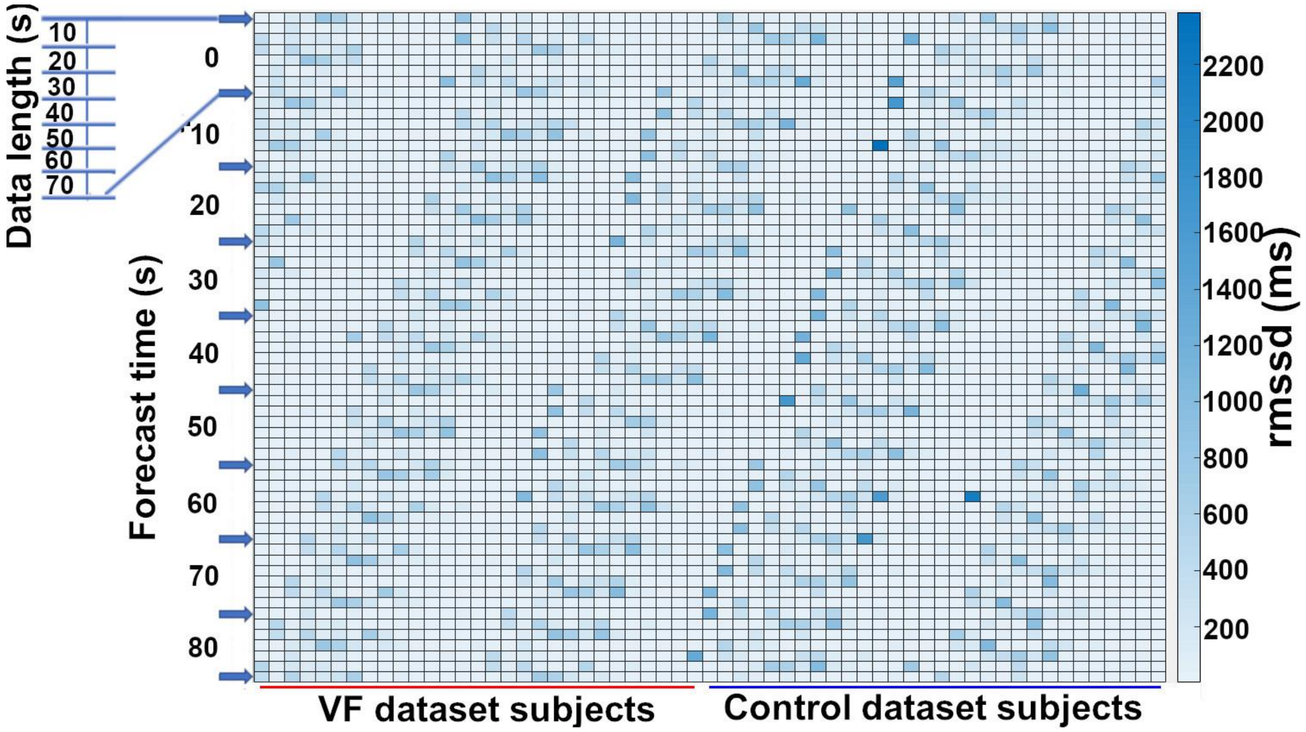

Figure 3. Proportion of interval differences of successive RR intervals greater than 50 ms (pnn50)

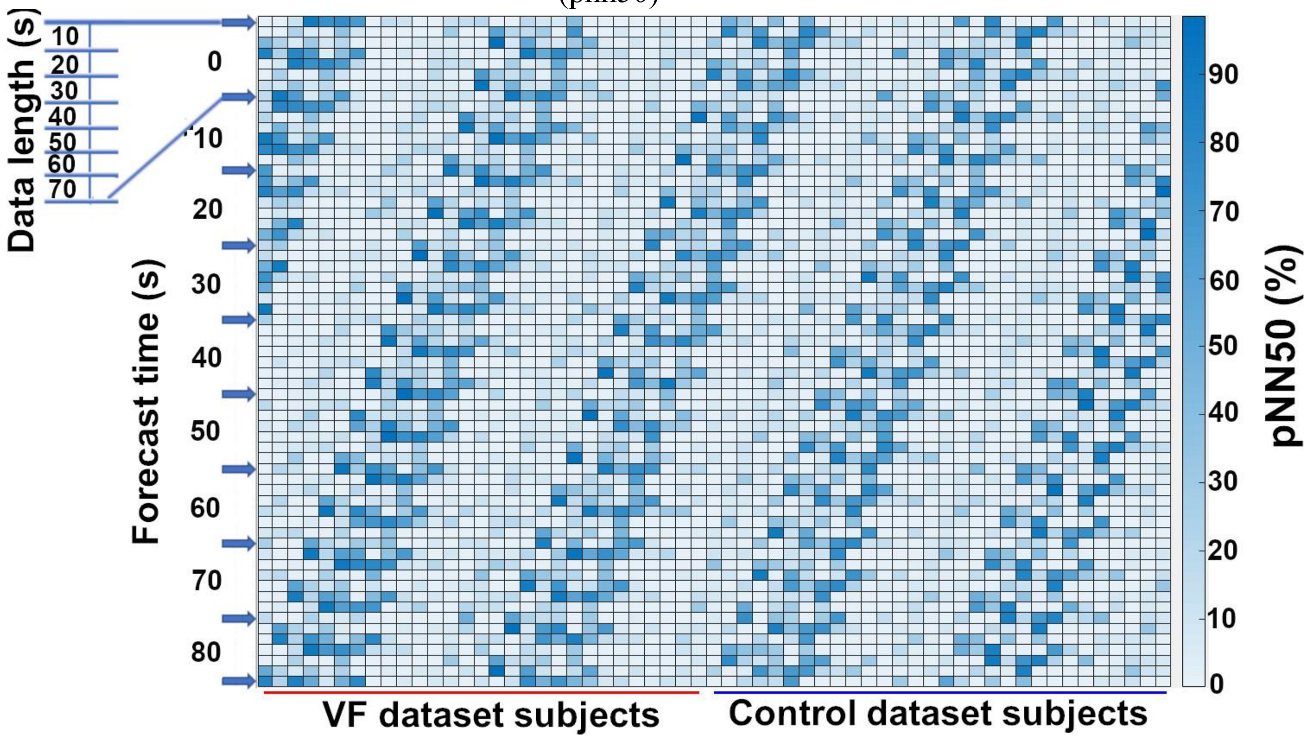

Figure 4. Square root of the mean squared differences of successive NN (RMSSD).

Frequency domain features for HRV

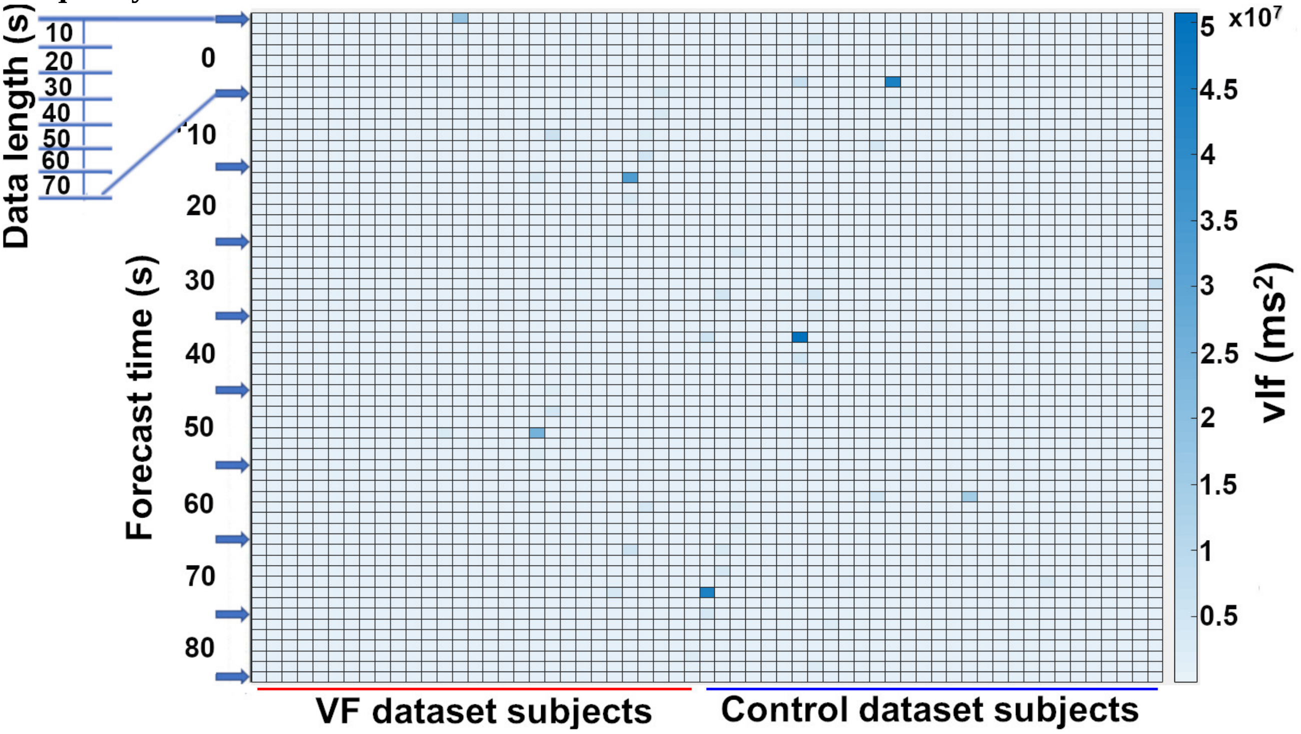

Figure 5. Power in very low frequency range (0–0.04 Hz) (VLF).

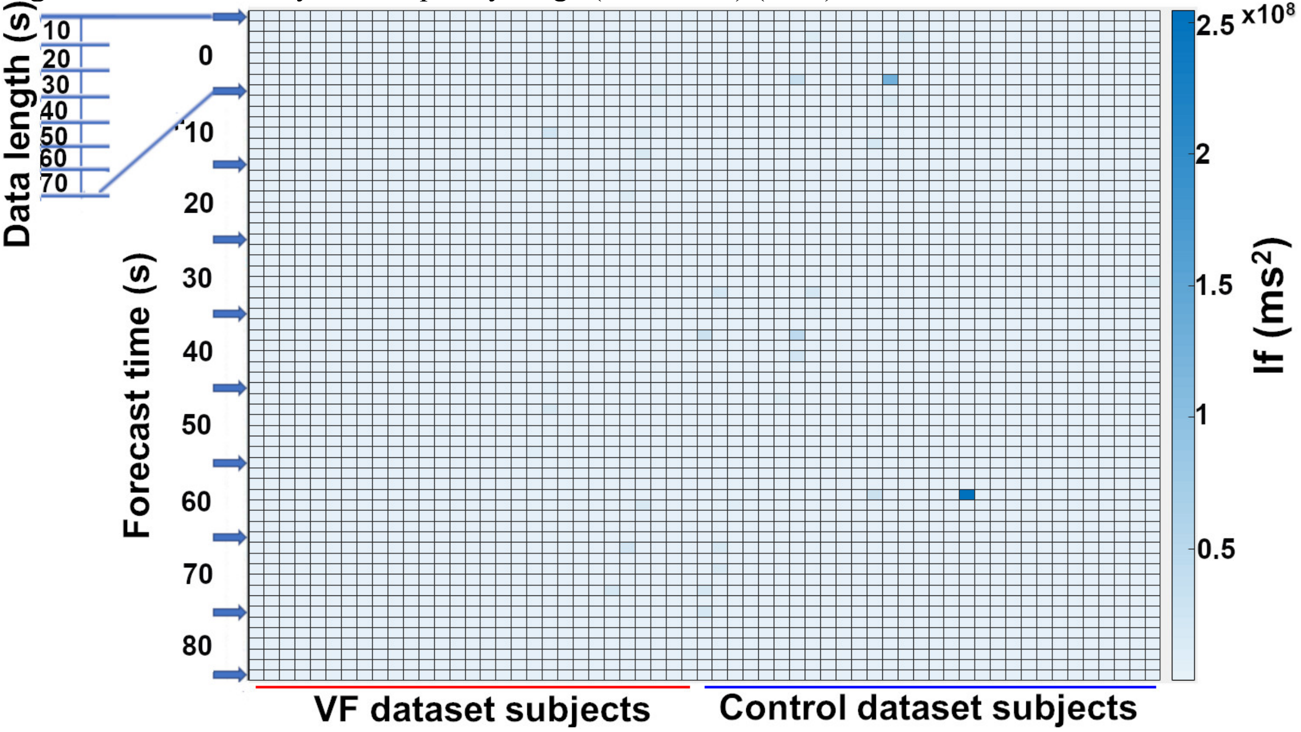

Figure 6. Power in low frequency range (0.04–0.15 Hz) (LF).

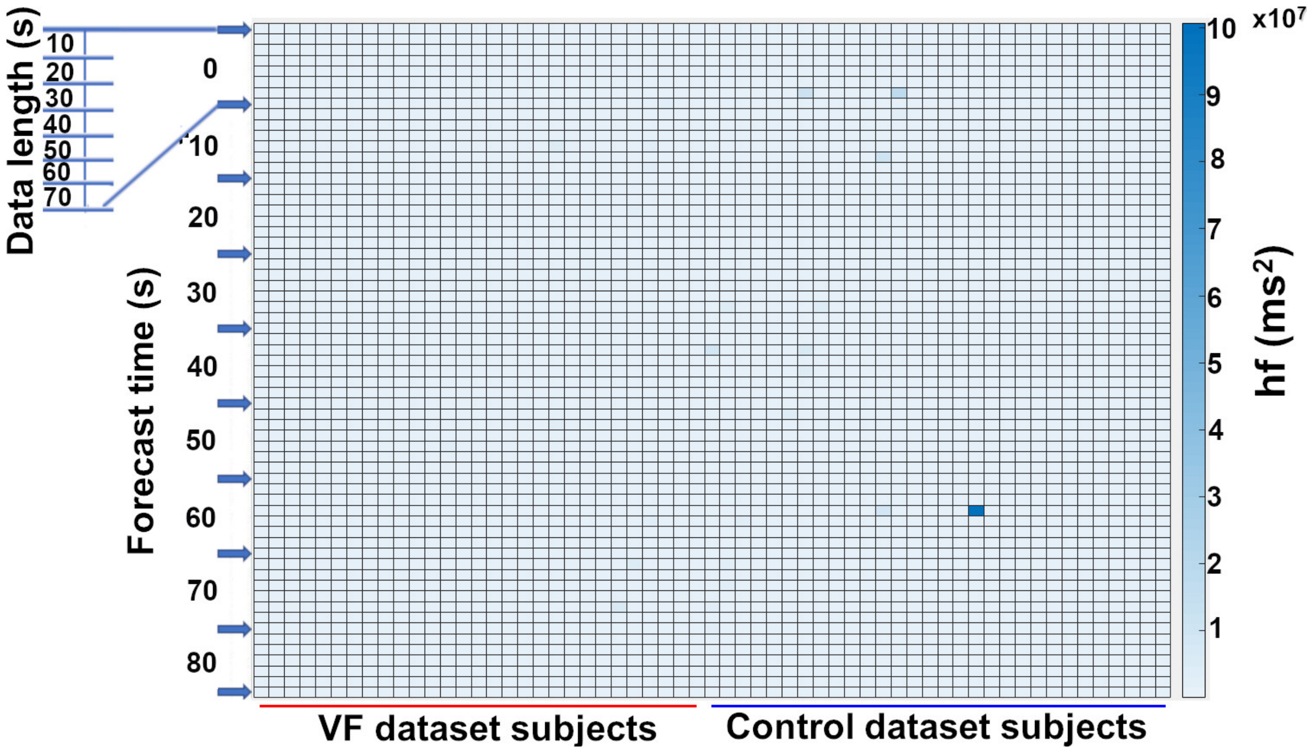

Figure 7. Power in high frequency range (0.15–0.4 Hz) (HF).

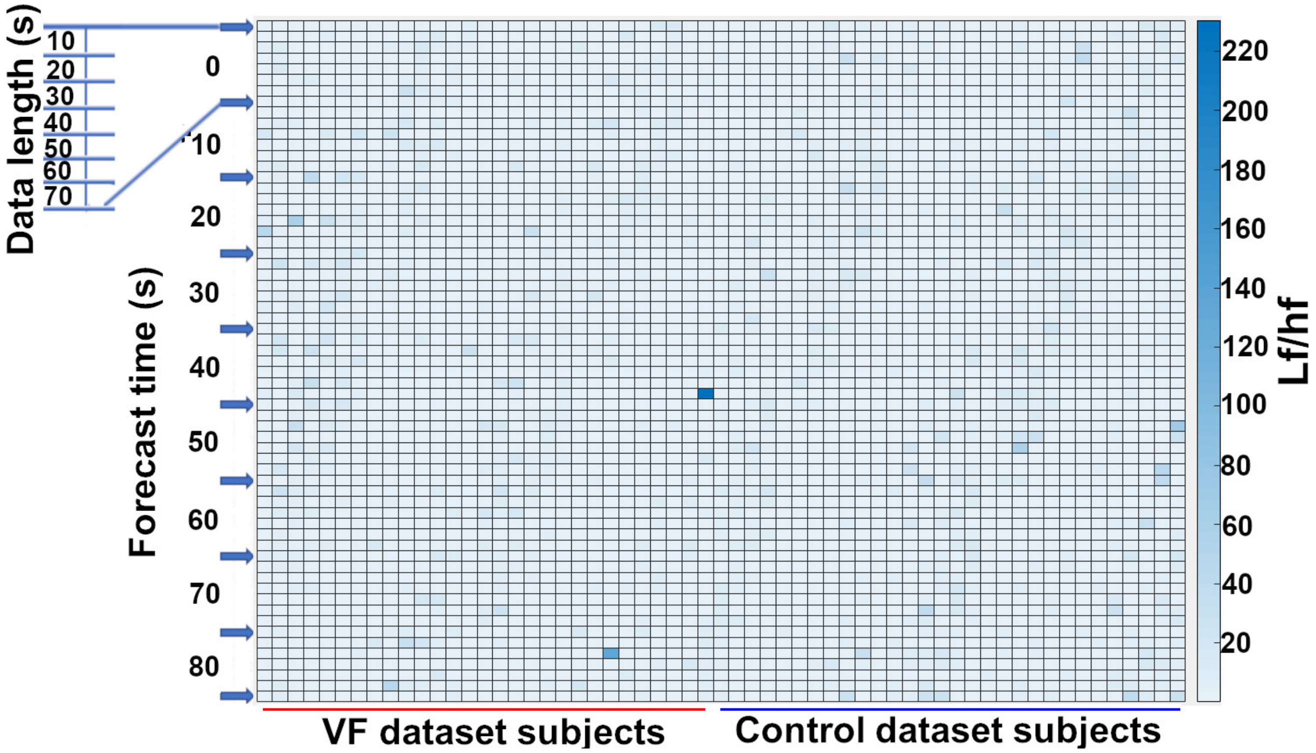

Figure 8. Ratio of LF to HF (LF/HF).

Poincare nonlinear features for HRV

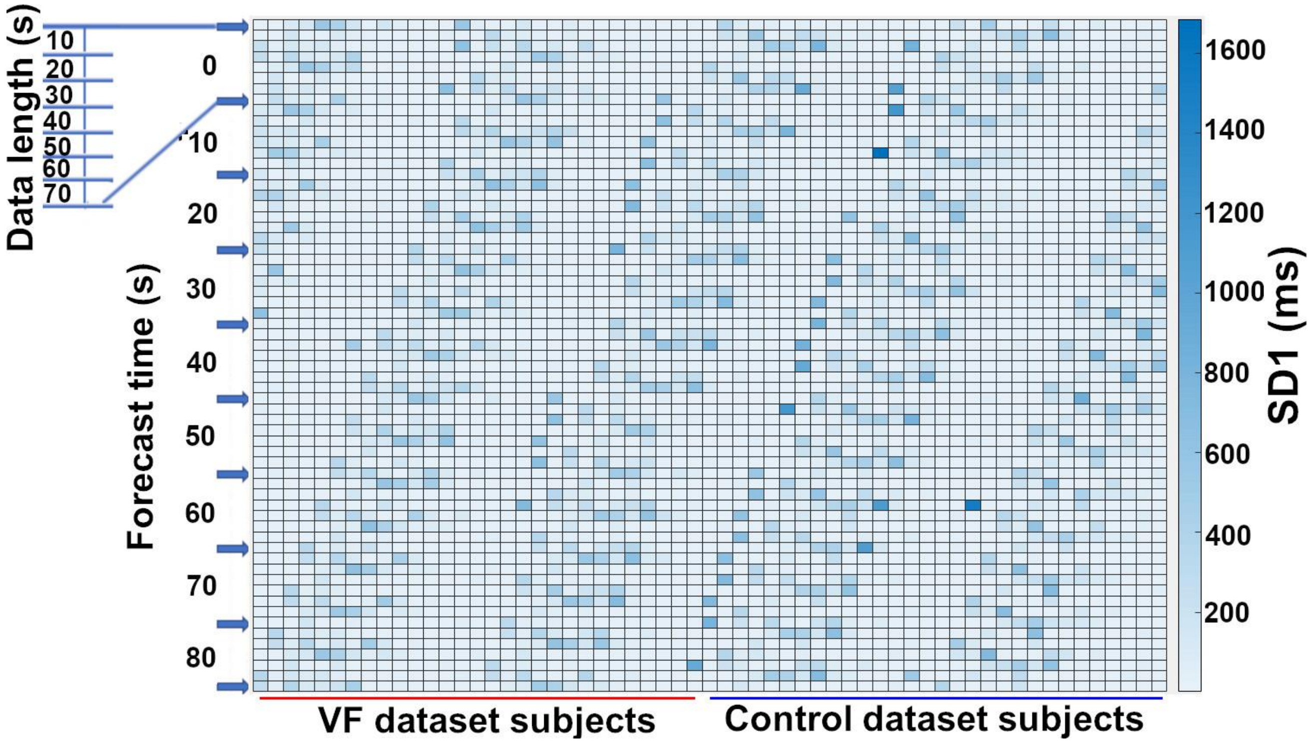

Figure 9. Standard deviation of points perpendicular to the axis of line of identity (SD1).

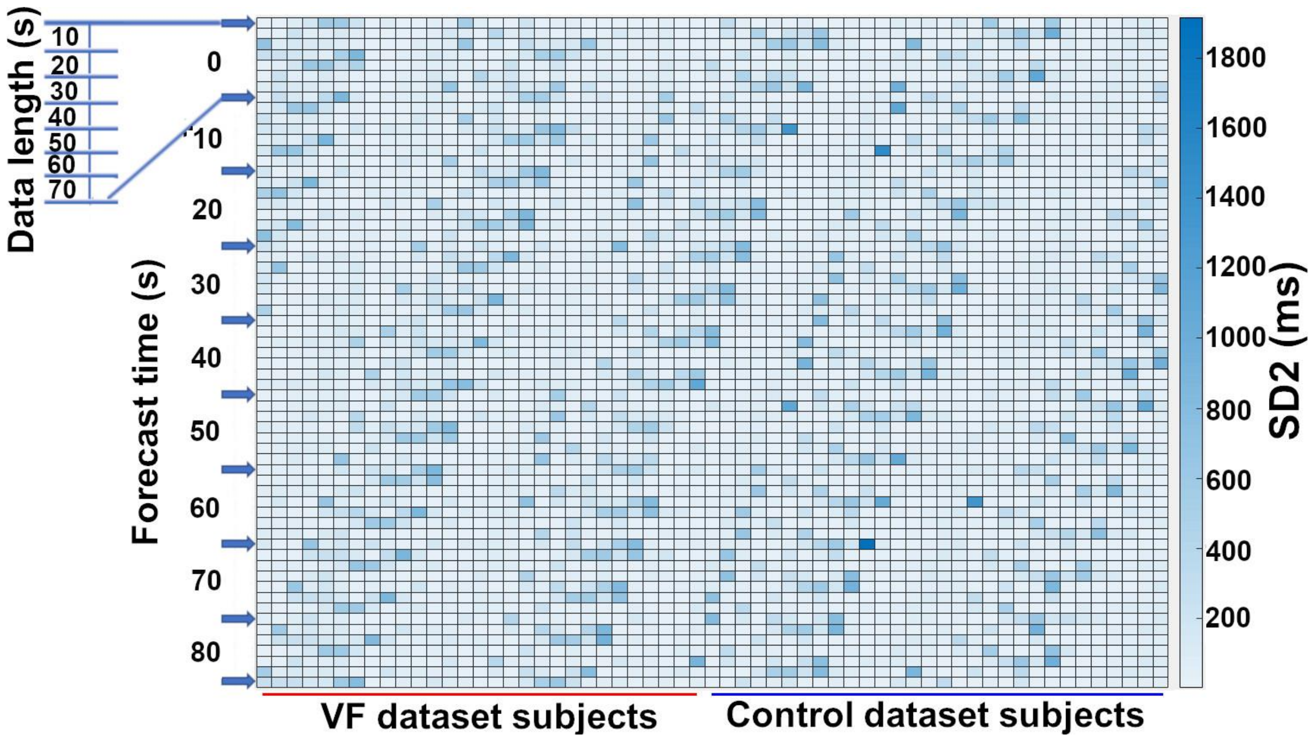

Figure 10. Standard deviation of points along the axis of line of identity (SD2).

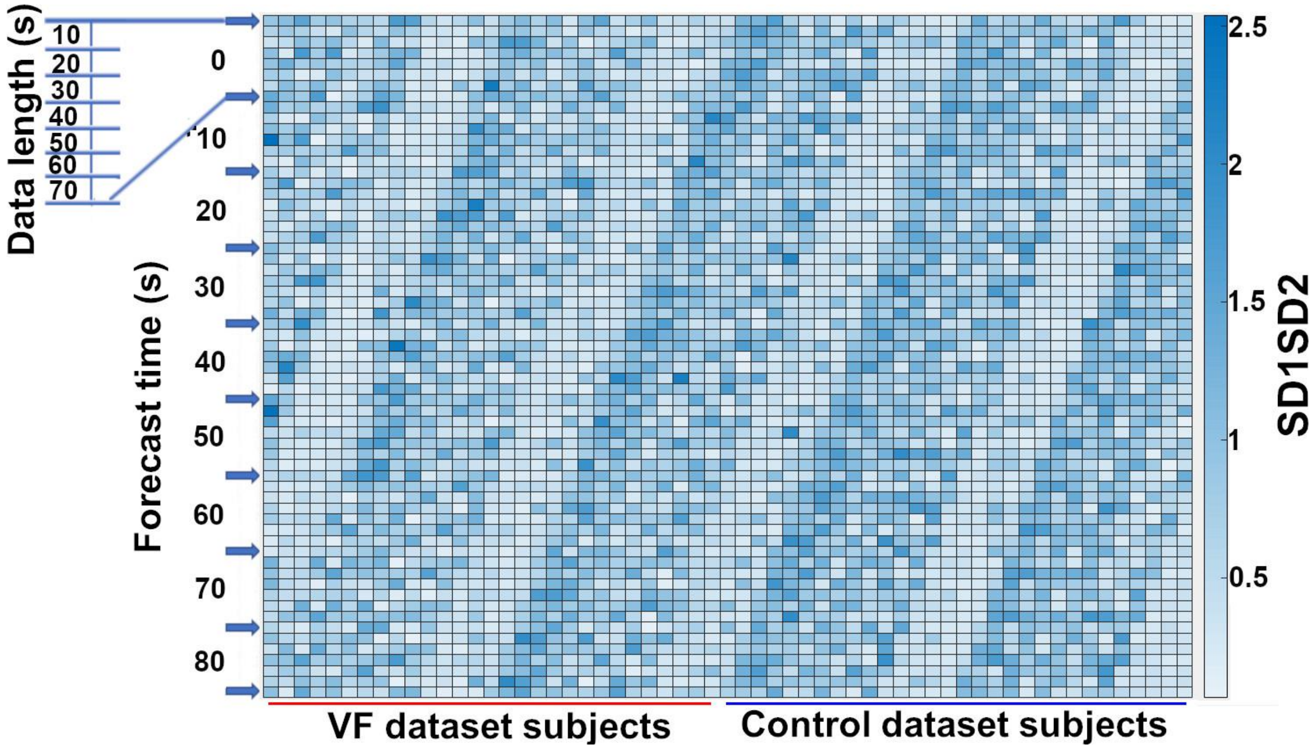

Figure 11. Ratio of SD1 to SD2 (SD1/SD2).
